# Plötzlich auftretende Doppelbilder – eine interdisziplinär behandelte Komplikation

**DOI:** 10.1007/s00106-024-01450-x

**Published:** 2024-03-18

**Authors:** C. Schmit, A. Runge, A. Jöbstl, G. Widmann, J. Schmutzhard, B. Hofauer

**Affiliations:** 1 Universitätsklinik für Hals‑, Nasen- und Ohrenheilkunde, Medizinische Universität Innsbruck, Anichstraße 35, 6020 Innsbruck, Österreich; 2 Universitätsklinik für Radiologie, Medizinische Universität Innsbruck, Innsbruck, Österreich

## Falldarstellung

### Anamnese

Unser 15-jähriger Patient stellte sich mit seit zwei Tagen bestehender Abgeschlagenheit sowie nun neu aufgetretenen Doppelbildern bei dem Blick nach rechts auf der Hals-Nasen-Ohren-Klinik vor. Der junge Kampfsportler berichtete, vor vier Wochen Ohrenschmerzen rechts gehabt zu haben.

Ein rezentes Trauma wurde seinerseits verneint. Zum Zeitpunkt der Vorstellung wurden otologische Beschwerden, insbesondere Otalgie, Otorrhö, Schwindel, Tinnitus oder eine subjektive Hörminderung verneint.

Auch rhinosinusitische Beschwerden, Fieber oder Kopfschmerzen wurden verneint. Sonstige Vorerkrankungen waren nicht bekannt, laut seiner Mutter hatte er in der Kindheit nicht unter rezidivierenden Mittelohrentzündungen oder Paukenergüssen gelitten.

### Klinische Untersuchung

In der Ohrmikroskopie zeigte sich das Trommelfell auf der rechten Seite differenziert und reizlos, die Pauke eindeutig lufthaltig, und das Valsalva-Manöver zeigte sich prompt positiv.

**Abb. 1:**
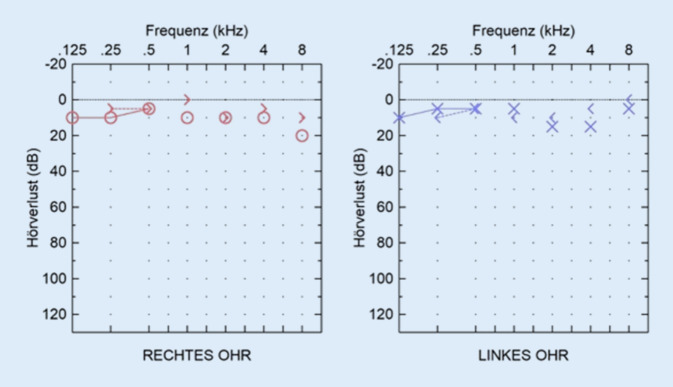
Tonaudiogramm. Es zeigte sich ein annähernd seitensymmetrischer Befund ohne Schallleitungs- oder Schallempfindungskomponente

Ein durchgeführtes Tonaudiogramm zeigte einen regelrechten, altersentsprechenden Befund (Abb. 1), die Impedanz entsprach beidseits einem Typ A.

Der restliche HNO-Status ergab keinen pathologischen Befund.

In der orientierenden neurologischen Untersuchung zeigte sich eine Augenmotilitätsstörung, entsprechend einer isolierten Parese des N. abducens (VI) auf der rechten Seite mit resultierenden horizontalen Doppelbildern beim Blick nach rechts. Die restlichen Hirnnerven zeigten sich in der klinischen Prüfung intakt.

### Diagnostik und weiteres Prozedere

Die unmittelbar eingeleiteten diagnostischen Maßnahmen beinhalteten eine Computertomographie (CT) des Schädels, eine Liquorpunktion sowie eine tiefgreifende laborchemische Untersuchung.

Die durchgeführte Liquoruntersuchung stellte sich unauffällig dar, und in der laborchemischen Untersuchung zeigten sich lediglich eine minimale Leukozytose mit 11,49 G/l, das C‑reaktive Protein (CRP) bei 0,95 mg/dl.

**Abb. 2:**
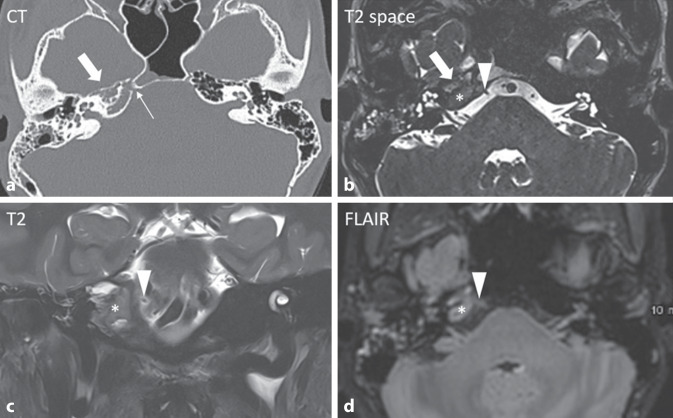
**a **Die hochauflösende axiale CT-Bildgebung zeigt eine lytische Destruktion der Knochentrabekel des petrösen Apex sowie dessen Obliteration ohne Expansion (*dicker Pfeil*). **b–d **In der Magnetresonanztomographie (MRT) ergibt sich korrelierend das Bild eines inflammatorischen Pseudotumors im petrösen Apex (*Stern*). **a **Der N. abducens kann computertomographisch nicht direkt visualisiert werden, aber sein Verlauf im Dorello-Kanal durch die destruierte Felsenbeinspitze ist darstellbar (*dünner Pfeil*). **b–d **Die axialen T2-Space‑, FLAIR- und die koronaren T2-Bilder erlauben eine direkte Visualisierung des N. abducens (*Pfeilspitze*) angrenzend an die entzündlichen Veränderungen

Die hochauflösende axiale CT-Bildgebung zeigte eine Obliteration der Felsenbeinspitze mit lytischer Destruktion der Knochentrabekel. Die zerebralen sowie Mittelohr- und Innenohrstrukturen zeigten sich unauffällig. Bildgebend zeigte sich der Verlauf des N. abducens im Dorello-Kanal durch die destruierte Felsenbeinspitze (Abb. 2).

In weiterer Folge erfolgte noch eine zerebrale Magnetresonanztomographie (MRT), hier ergab sich korrelierend das Bild eines inflammatorischen Pseudotumors im petrösen Apex. Der N. abducens zeigte sich unmittelbar angrenzend an die entzündlichen Veränderungen (Abb. 2 und 3).

**Abb. 3:**
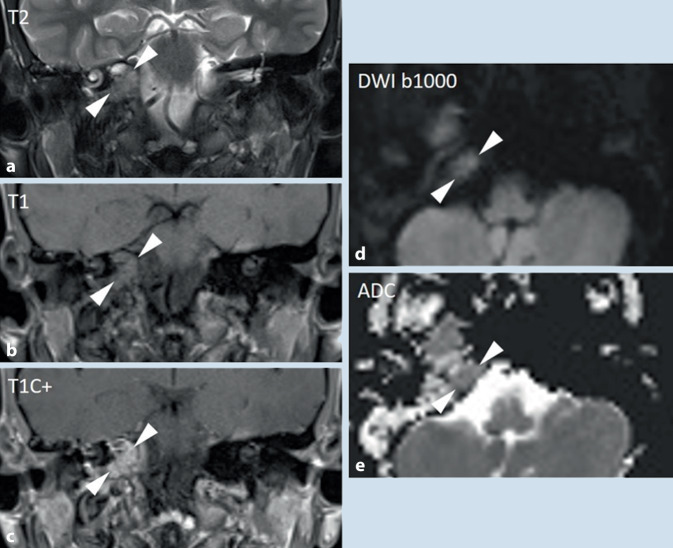
**a **T2-gewichtet zeigt sich eine geringe Hyperintensität des petrösen Apex mit isointensem Signal in **b **T1 sowie homogenem Kontrastmittelenhancement in **c **T1C+. Es besteht keine Diffusionsstörung bei nur geringer Hyperintensität in der **d **DWI (Folge des T2-Shine-Through-Effekts) und ohne relevanten Abfall der **e **Apparent Diffusion Coefficient-Werte (ADC).

Entsprechend der Verdachtsdiagnose erfolgte die sofortige Einleitung einer antibiotischen Therapie sowie eine interdisziplinäre Rücksprache mit den Kolleg*innen der Neurochirurgie.

## Wie lautet Ihre Diagnose?

**Diagnose:** Gradenigo-Syndrom

Das Gradenigo-Syndrom ist eine Komplikation der akuten Otitis media.

### Therapie und Verlauf

In unserem Fall wurde entsprechend der Empfehlungen eine intravenöse antibiotische Therapie mit Ceftriaxon und Metronidazol eingeleitet. Die Möglichkeit einer zusätzlichen chirurgischen Sanierung der beteiligten Felsenbeinspitze wurde interdisziplinär mit den Kolleg*innen der Neurochirurgie besprochen.

Aufgrund des unauffälligen ohrmikroskopischen und audiometrischen Befundes wurde sich für den Hör-, und Gleichgewichtserhaltenden operativen Zugang nach Kawase zur Felsenbeinspitze im Falle einer erforderlichen chirurgischen Sanierung entschieden.

Jedoch konnte aufgrund der, im weiteren stationären Verlauf, unter konservativer Therapie rasch regredienten Abduzensparese, des jungen Alters des Patienten sowie dessen stabilen Allgemeinzustandes auf eine operative Sanierung verzichtet werden.

Eine im Verlauf durchgeführte Gesichtsfeldmessung zeigte eine Normalisierung der Okulomotorik.

## Diskussion

Bei gering ausgelenkten Entzündungsparametern, nicht entzündlich verändertem Liquor und einem bis auf die N.-abducens-Parese bestehenden stabilen Allgemeinzustand kann eine fulminante Meningitis oder Enzephalitis als unwahrscheinlich betrachtet werden.

Die bildgebend dargestellte lytische Destruktion der Knochentrabekel im petrösen Apex mit Obliteration und Kontrastmittelaufnahme, jedoch ohne expansives Wachstum spricht für einen entzündlichen Prozess [1]. Auch differenzialdiagnostisch zu erwägende raumfordernde Prozesse, wie das Cholesteringranulom und das Cholesteatom, können aufgrund der Signalintensität, des Kontrastmittelenhancements sowie des Fehlens einer Diffusionsrestriktion in den entsprechenden MRT-Sequenzen ausgeschlossen werden. Auch kann eine maligne Läsion aufgrund der fehlenden Expansion und Diffusionsrestriktion ausgeschlossen werden (Abb. 3; [1]).

In Anbetracht der Anamnese, des klinischen Befundes sowie insbesondere der bildgebenden Befunde wurde die Verdachtsdiagnose eines Gradenigo-Syndroms (GS) gestellt.

Das im Jahr 1904 erstbeschriebene und nach dem Entdecker Giuseppe Gradenigo benannte Syndrom beschreibt eine Trias aus Otorrhö, einseitigen Gesichtsschmerzen und horizontalen Doppelbildern.

Ursächlich hierfür ist eine akute bakterielle Otitis media, in deren Folge eine fortgeleitete Entzündung der Felsenbeinspitze auftritt. Eine ausreichend pneumatisierte Pyramidenspitze stellt hierfür einen prädisponierenden Faktor dar [2].

Die in etwa der Hälfte der Fälle auftretende Parese des VI. Hirnnervs ist aufgrund des prädisponierenden Nervenverlaufs als toxisch zu werten. Eine Mitbeteiligung des V. Hirnnervs mit charakteristischem Trigeminusschmerz ist durch eine entzündliche Affektion des Ganglion Gasseri bedingt [3]. Durch die inzwischen standardmäßige antibiotische Therapie der akuten bakteriellen Mittelohrentzündung handelt es sich um eine seltene Komplikation [4]. Nur in etwa 1/5 der Fälle tritt das klassische Vollbild auf [5].

Die auslösende Mittelohrentzündung kann zum Zeitpunkt des Auftretens des Gradenigo-Syndroms bereits abgeheilt sein [3]. In der Literatur werden zeitliche Abstände zwischen Otitis media und GS von bis zu drei Monaten beschrieben [2].

Im vorgestellten Fall bestand zu keinem Zeitpunkt eine Otorrhö. Jedoch berichtete der Patient über eine einen Monat zurückliegende Otalgie, hinweisend auf abgelaufene Mittelohrentzündung, welche jedoch keine spezifische Therapie nach sich zog.

Eine breite antibiotische Therapie stellt in der Behandlung des Gradenigo-Syndroms den Grundpfeiler dar. Zu den häufigsten Keimen gehören unter anderem Pseudomonaden, Streptokokken sowie Staphylokokken [5]. Folglich muss die antibiotische Therapie das grampositive, das gramnegative und das anaerobe Spektrum abdecken.

Bei ausbleibender klinischer Besserung unter dem konservativen Therapieschema wird eine chirurgische Sanierung notwendig. Hierbei ist ein besonderes Augenmerk auf die Drainage der Pyramidenspitze zu legen. Ein transcochleärer oder ein transtemporaler Zugang stellen mögliche operative Zugangswege dar [3].

## Fazit für die Praxis

- Komplikationen der Otitis media sind seit der Einführung der antibiotischen Therapie selten zu beobachten.
- Die klassische Trias des Gradenigo-Syndroms tritt nur in seltenen Fällen auf.
- Der zeitliche Rahmen zu der vorangegangenen Mittelohrentzündung ist sehr variabel.
- Abstände bis zu drei Monate werden beschrieben.
- Die bildgebenden Verfahren stehen im Zentrum der diagnostischen Maßnahmen.
- Grundpfeiler der Behandlung ist eine breite antibiotische Therapie.
- Zusätzlich kommen bei nicht ausreichendem Ansprechen auf die konservative Therapie chirurgische Optionen infrage.
